# A multidisciplinary Delphi consensus to define evidence-based quality indicators for diabetic foot ulcer care

**DOI:** 10.1093/eurpub/ckad235

**Published:** 2024-01-26

**Authors:** Flora Mbela Lusendi, An-Sofie Vanherwegen, Frank Nobels, Giovanni Arnoldo Matricali

**Affiliations:** Department of Epidemiology and Public Health, Health Services Research, Sciensano, Brussels, Belgium; Department of Development and Regeneration, KU Leuven, Leuven, Belgium; Department of Epidemiology and Public Health, Health Services Research, Sciensano, Brussels, Belgium; Department of Internal Medicine-Endocrinology, Multidisciplinary Diabetic Foot Clinic, Onze-Lieve-Vrouw Ziekenhuis Aalst, Aalst, Belgium; Department of Development and Regeneration, KU Leuven, Leuven, Belgium; Multidisciplinary Diabetic Foot Clinic, University Hospital Leuven, Leuven, Belgium

## Abstract

**Background:**

Valid measures to assess quality of care delivered to patients with diabetes suffering from diabetic foot ulcer (DFU) are scarce. This study aimed to achieve consensus on relevant and feasible quality indicators (QIs) among stakeholders involved in DFU care and was conducted as the second part of a Belgian QI selection study that sought to identify QIs for DFU care.

**Methods:**

A stakeholder panel, including caregivers from primary care and specialized disciplines active in diabetic foot care as well as a patient organization representative, was recruited. By using the RAND/UCLA Appropriateness Method, stakeholders were asked to rate a list of 42 candidate evidence-based indicators for appropriateness through a 9-point Likert scale. QIs were classified based on the median ratings and the disagreement index, calculated by the inter-percentile range adjusted for symmetry.

**Results:**

At the end of a three-phase process, 17 QIs were judged as appropriate. Among them, five were not previously described, covering the following topics: integration of wound care specialty in the multidisciplinary team, systematic evaluation of the nutritional status of the patient, administration of low-density lipoprotein-cholesterol lowering medication and protocolized care (implementation of care and prevention management protocols).

**Conclusions:**

The identified evidence-based QIs provide an assessment tool to evaluate and monitor quality of care delivered to DFU patients. Future research should focus on their complementarity with the existing QIs and their implementation in clinical practice.

## Introduction

A quality indicator of care (QI) is defined as a measurable aspect of care (structure, process or outcome) for which there is sufficient evidence and/or consensus that it can be used to evaluate quality of care and its evolution.^1^^,^^2^ Two main steps have been identified for the development of QIs: the collection of existing knowledge for the creation of potential QIs and the establishment of a consensus on the proposed QIs to be used.^3^^,^^4^ The first step consists of synthesizing the scientific literature and/or supplemental sources (e.g. grey literature). However, for many areas of healthcare, the available evidence challenges the development of QIs. This may be due to limited or inconclusive scientific evidence or lack of evidence for the specific population of interest (with the need to extrapolate results from other patient populations).^4^ These challenges can largely be addressed with the use of a consensus method as a second step, which constitutes the most common formal approach to make decisions, generate ideas or establish a ranking when scientific evidence is inconclusive.^5^ It is based on the involvement of a group of stakeholders, who discuss the topic taking into account different perspectives and providing a more nuanced input, considering clinical relevance and feasibility.

Diabetic foot ulcer (DFU) is a multifactorial chronic condition with a global prevalence of 6,3% among people with diabetes^6^ and with a huge impact on quality of life^7^ and healthcare expenditure.^8^ The condition is an advanced stage diabetes complication occurring in multimorbid patients with long diabetes duration, which makes treatment complex. To tackle this complexity, care is often organized in a multidisciplinary way, including endocrinologists, orthopedic and vascular surgeons, podiatrists, diabetes nurses, wound care nurses and shoe technicians.^9^^,^^10^ To optimize this complex care, systems of quality evaluation and monitoring have been implemented in some countries. For this purpose, QIs have been developed and implemented in the frame of national audit-feedback initiatives organized in collaboration with diabetic foot services.^11^ For example, in Belgium, diabetic foot experts decided, based on their clinical experience, to focus on certain processes and outcomes of care as well as the patient-level parameters that might affect these. However, the QIs used up to now present some limitations. They have not been identified in an exhaustive manner and thus might not consider all aspects of care, nor all interventions that may provide opportunities to improve DFU outcomes. Furthermore, not all DFU stakeholders were represented during the indicator selection and a formal selection methodology was not applied.

The high societal impact and the complex management of DFU warrant efforts to address the identified limitations of existing QIs. The present study aims to describe a selection of evidence-based QIs for DFU care by a multidisciplinary stakeholder panel consisting of the previously mentioned careholders, using a formal consensus method. We do not aim to displace well-established QIs but rather to reinforce existing and identify new evidence-based quality indicators. The proposed list of candidate indicators was established based on a systematic and open-minded (not limited to guidelines) search of the literature and focused on structure and process QIs (manuscript submitted for publication). This article describes the second key step in developing a set of evidence-based QIs for Diabetic Foot Clinics (DFCs).

## Methods

### Literature review and identification of candidate quality indicators

We conducted a scoping review of the literature to identify available evidence-based interventions that could be used as a process or structure indicator to assess quality in DFCs (manuscript submitted for publication). In summary, we performed structured searches of four electronic databases (PubMed, Embase, CINAHL and Cochrane Library) for publications between database inception and 3 March 2020. We selected studies reporting interventions related to organization or delivery of care based on defined eligible criteria. From the 322 clinical studies included, 37 process indicators and 5 structure indicators were generated.

The set of 42 candidate indicators covered the following diabetic foot care domains: organization of care, wound healing interventions, peripheral artery disease (PAD), offloading and secondary prevention (Supplementary table S1).

### Selection of stakeholder panel

Panel members were recruited to represent the disciplines corresponding to the staff involved in recognized Belgian DFCs,^11^^,^^12^ including diabetologist, orthopedic surgeon, vascular surgeon, podiatrist, diabetes nurse and/or wound care nurse and shoe technician. Besides these disciplines, the following stakeholder groups were considered: representative of the diabetes patient organization, general practitioner and employee of the National Institute for Health and Disability Insurance (NIHDI), which is the national organization for social security and reimbursement.^13^ Belgian multidisciplinary DFCs and the national general practitioner network were contacted. We aimed to include one Dutch-speaking and one French-speaking representative for each selected discipline to represent the main Belgian linguistic communities. English was used as a common language since it constitutes the universal form of communication in science. To those who expressed their interest, a copy of the curriculum vitae was requested. Candidates were selected based on their expertise and representativeness of their stakeholder group, their availability at the meeting date and their command of English. The panel consisted of four diabetologists, two vascular surgeons, three podiatrists (of which one also had a shoe technician background), two orthopedic surgeons, one general practitioner, one diabetes nurse and one employee of NIHDI. The general practitioner was the chairperson of the Diabetes Association where both patients and professionals join forces and thus was committed to ensure that the patient voice is taken into account. Stakeholders were financially compensated with a gift voucher (retribution).

### Selection of quality indicators based on the RAND/UCLA appropriateness methodology

**Figure 1.**
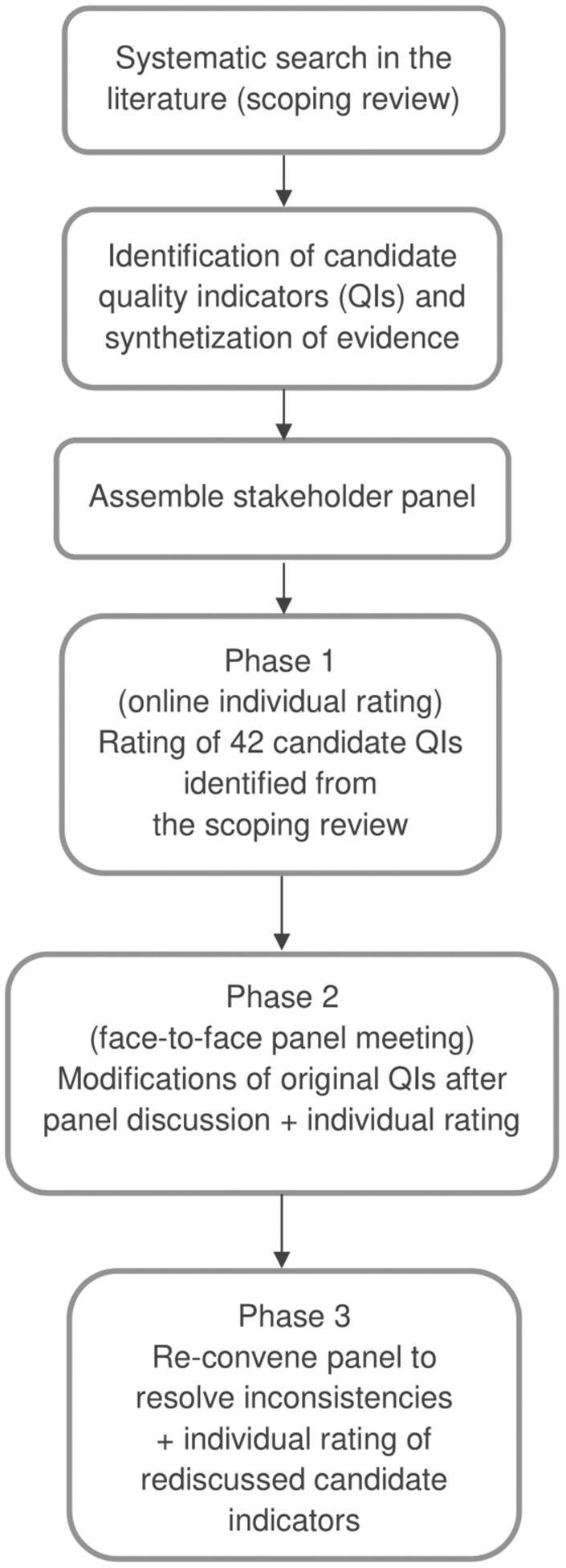
Process flowchart of the RAND/UCLA appropriateness method.

The stakeholder panel followed the guidelines of the formal consensus process RAND/UCLA Appropriateness Method (RAM)^14^ to select evidence-based QIs for DFCs. It consists of two rating phases, with a face-to-face meeting between the rating phases. The approach relies on evidence-based medicine to guide stakeholders, stimulate their discussions and facilitate the collective opinion. The process flowchart of the RAND/UCLA appropriateness method is outlined in figure 1.

In the first phase, panelists received an online survey along with the following documents: a summary document that provided information about the study background and the used methodology, a booklet describing each indicator, its characteristics and its synthesized supporting scientific evidence, the references of publications used to produce the candidate QIs and a glossary. The survey was administered through LimeSurvey. A personal access code (token) was provided to each panelist. Each panelist was asked to rate the 42 candidate indicators on their appropriateness by using the RAND/UCLA 9-point Likert scale, defined as follows: 1 = highly inappropriate, 5 = intermediate rating; benefits and harms are about equal or the rater cannot make the judgement, 9 = highly appropriate. Next, ratings of this first phase were analyzed and summarized for the second phase. Based on the RAND/UCLA method, an indicator was classified into three levels of appropriateness, using the following definitions: appropriate (panel median of 7–9, without disagreement), uncertain (panel median of 4–6 or any median with disagreement), inappropriate (panel median of 1–3, without disagreement). The disagreement was quantified by using the RAND ‘Disagreement Index’ (DI).^14^^,^^15^ The DI is defined as the ratio of two major elements: the inter-percentile range (difference between 25th and 75th percentile) and the inter-percentile range adjusted for symmetry (dispersion of scores). When DI is ≤1.0, no disagreement exists among the panelists. The lower the DI, the lower the level of disagreement (i.e. the higher the level of agreement). A personalized panelist rating sheet was prepared for the second phase in order to give the panelists the opportunity to discuss their ratings, in light of the information concerning the other panelists’ ratings. For each indicator, the following items were indicated: the panelist’s own ratings of phase 1 as well as the distribution of scores from the other panelists (individual panelist’s ratings were kept confidential), the panel median score, its associated level of appropriateness and the level of disagreement.

The second phase consisted of a face-to-face meeting under the leadership of a moderator. A summary of phase 1 results was presented, focusing on the candidate indicators for which there was disagreement. The panelists were encouraged to share comments, to reflect on their own ratings from phase 1 and were given the opportunity to modify the formulation of the original indicators listed or to propose new indicators. During the meeting, each panelist rated the appropriateness of each indicator again, taking into account possible modifications that were proposed, regardless of whether a consensus was reached or not in phase 1.

After examining the ratings of phase 2, inconsistencies were observed. Based on the recommendations from the RAND/UCLA methodology for resolving inconsistencies,^14^ the panel was convened at an additional meeting (phase 3) to discuss the issues. During this third meeting, which was organized remotely, panelists expressed their opinions on the observed issues. Afterwards, panelists were asked to rate the appropriateness of the re-discussed indicators by considering the exchanges that took place during the online discussion. For this purpose, an online survey was sent along with a report describing the meeting discussion. The scores assigned during phases 2 and 3 were used to determine a final set of QIs. Only the indicators with a median rating of ≥7 and with no disagreement based on DI were selected as QIs for DFCs.

## Results

**Figure 2.**
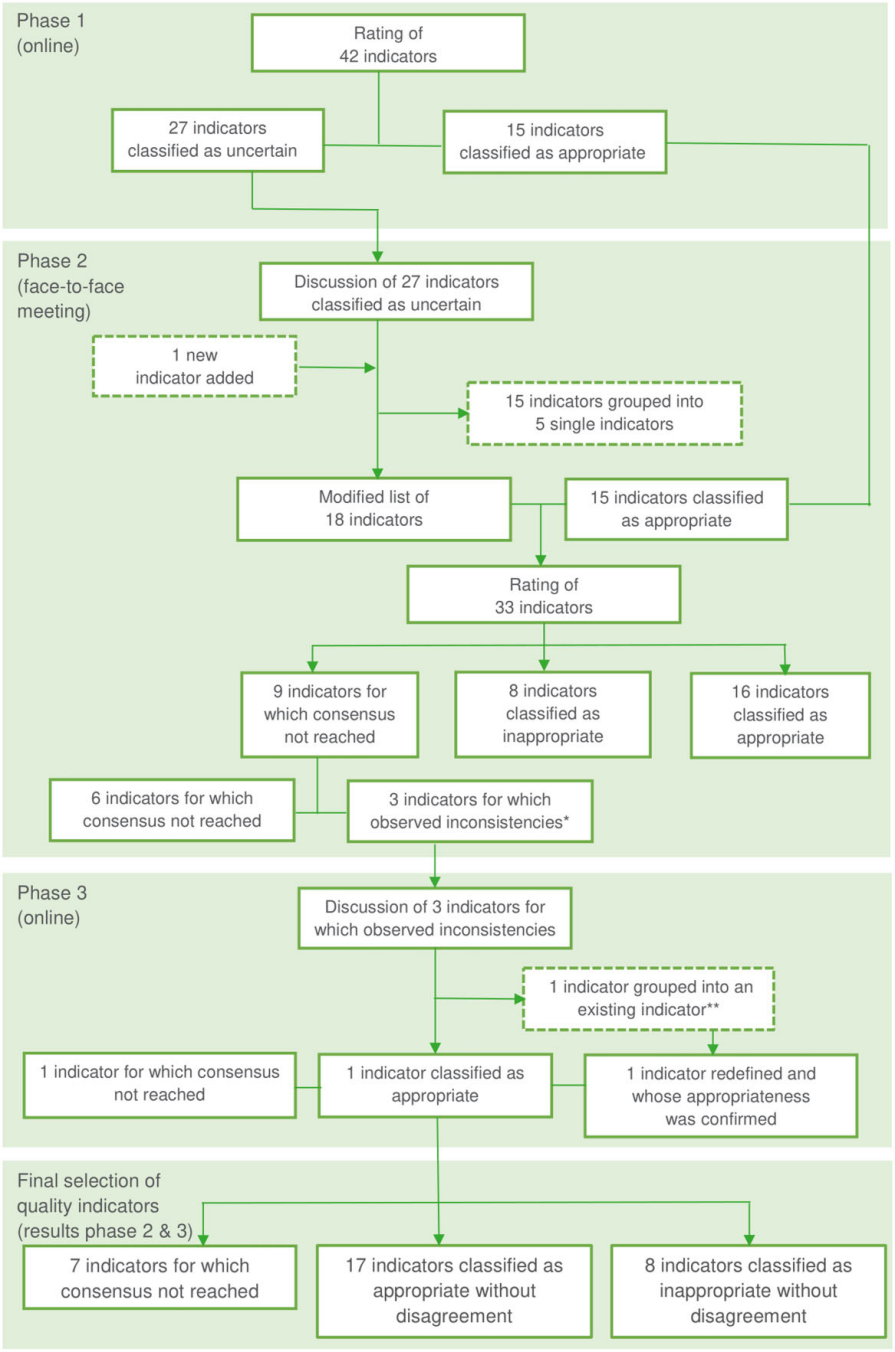
The three-step rating of the quality indicators. *Misunderstandings and unexplained shifts in the appropriateness classification. **The formulation of indicator A.6 was modified to integrate indicator A.7 (Proportion of people with a diabetic foot ulcer receiving multidisciplinary foot care with an integrated skin graft specialty)

The evaluation of candidate QIs occurred in three distinct phases. In total, 13 panelists participated in the full three-phase rating process. The shoe technician and the employee of NIHDI completed the first phase but were not able to participate in the next phases. Only the shoe technician could be replaced. An overview of the different rating steps of the QIs can be found in figure 2.

### Phase 1: rating of 42 candidate indicators

At the end of phase 1, from the 42 candidate indicators, 27 (64%) were classified as uncertain and 15 (36%) were classified as appropriate. The appropriate indicators included four indicators addressing the domain of organization of care (A.1–A.2–A.3–A.5), four addressing the domain of wound healing (B.6–B.11b–B.12b–B.12c), one addressing the domain of PAD (C.1a), two addressing the domain of offloading (D.1–D.2) and four addressing the domain of secondary prevention (E.1–E.2a–E.2b–E.3).

### Phase 2: face-to face meeting

During the face-to-face meeting in phase 2, the discussion focused on the 27 indicators, which were classified as uncertain after the first phase. Among this set of 27 indicators, the stakeholders suggested to group, introduce or re-define a certain number of candidate indicators. A first suggestion was to re-define the indicators addressing non-biological dressings (B.1a–B.1b–B.1c) and bioengineered skin substitutes (B.2a–B.2b–B.2c–B.2d) into two therapy-specific indicators, which would measure the integration of the wound care specialty within the multidisciplinary team (A.6–A.7). A second suggestion was to group the three indicators addressing hyperbaric oxygen therapy (B.4a–B.4b–B.4c) into a single indicator without specifications on the target population (B.4). It was also suggested to combine the two indicators (B.9a–B.9b) addressing nutritional supplementation into a single indicator covering the evaluation of the nutritional status of the patient (B.9). Finally, a new indicator addressing mechanical debridement (B.11c) was introduced and the three indicators (C.1a–C.1b–C.1c) addressing the domain of PAD were combined into a single indicator (C.1). These changes were made during the meeting and resulted in a reduced list of 18 indicators. Between the discussions, stakeholders were invited to rate each of the 18 indicators and were given the opportunity to modify their ratings of phase 1 in light of these exchanges. As a result, a list of 33 indicators (i.e. the list of 18 indicators under discussion and the 15 indicators already rated as appropriate during phase 1) were assessed during phase 2.

Of the 33 candidate indicators, 16 indicators were classified as appropriate, 8 indicators as inappropriate and 9 as uncertain, which meant that a consensus could not be reached.

### Phase 3: resolving inconsistencies

Among the nine indicators classified as uncertain, there were three indicators (A.7–D.1–D.2) for which misunderstandings among the stakeholders and inconsistencies in appropriateness classification were suspected. For the first one (A.7), the analysis of the phase 2 results showed that the necessary modifications to the formulation of the indicator were not applied in the same way by all stakeholders. It was not clear if two distinct indicators, specific to the therapy used, had to be introduced. For the other two (D.1–D.2), we observed that a small shift in ratings had changed the appropriateness classification from appropriate in phase 1 to uncertain in phase 2. As recommended in the RAND/UCLA approach, an additional meeting (phase 3) was organized to discuss these inconsistencies. Considering the fact that the objective of the RAND/UCLA method is not to force the panel to a consensus, the six other indicators for which a consensus could not be reached after phase 2, were not discussed during phase 3. The assessment of the three ‘inconsistent’ indicators discussed and rated in phase 3 was as follows: the stakeholders could not reach a collective opinion for the indicator addressing the treatment with a knee-high offloading device (D.2), whereas the indicator addressing the treatment with a non-removable knee-high offloading device was classified as appropriate (D.1). In addition, the definition of the indicator covering the integration of a wound care specialty within the multidisciplinary team (A.6) proposed during phase 2, was clarified to combine knowledge on non-biological dressings and bioengineered skin substitutes. The appropriateness of that indicator (A.6) was confirmed, which resulted in the elimination of the therapy-specific indicator (A.7).

### Final selection of quality indicators

Considering the scores assigned during phases 2 and 3, the group of stakeholders classified 17 QIs as appropriate without disagreement (see table 1), 8 QIs as inappropriate without disagreement (Supplementary table S2) and 7 QIs as uncertain, meaning that a collective opinion could not be reached (Supplementary table S3).

**Table 1:** Final set of QIs classified as appropriate

No.	Indicator	Indicator type
Domain: Organization of care		
A.1	Proportion of people with a diabetic foot ulcer receiving multidisciplinary foot care	Structure
A.2	Proportion of people with a diabetic foot ulcer receiving multidisciplinary foot care with an integrated podiatric specialty	Structure
A.3	Proportion of people with a diabetic foot ulcer treated within the context of a care management program for diabetic foot	Structure
A.6	Proportion of people with a diabetic foot ulcer receiving multidisciplinary foot care with an integrated wound care specialty	Structure
Domain: Wound healing interventions		
B.6	Proportion of people with a non-healing diabetic foot ulcer treated with negative pressure wound therapy	Process
B.9	Proportion of people with diabetic foot ulcer for whom the nutritional status has been evaluated	Process
B.10a	Proportion of people with a non-healing diabetic foot ulcer treated with LDL-cholesterol lowering medication	Process
B.11c	Proportion of people with a non-healing diabetic foot ulcer treated with mechanical debridement	Process
B.12a	Proportion of people with a non-healing diabetic foot ulcer treated with major amputation	Process
B.12b	Proportion of people with a non-healing diabetic foot ulcer treated with bony surgical offloading	Process
B.12c	Proportion of people with a non-healing diabetic foot ulcer treated with soft tissue surgical offloading	Process
Domain: Peripheral artery disease (PAD)		
C.1	Proportion of people with a diabetic foot ulcer and inadequate perfusion treated with vascular surgery	Process
Domain: Offloading		
D.1	Proportion of people with a non-infected, non-ischemic plantar neuropathic diabetic foot ulcer treated with a non-removable knee-high offloading device	Process
Domain: Secondary prevention		
E.1	Proportion of people with a (history of) diabetic foot ulcer receiving patient education	Process
E.2a	Proportion of people with a history of peripheral neuropathy (PNP) receiving therapeutic footwear and/or custom-made insoles, or custom-made shoes	Process
E.2b	Proportion of people with a history of diabetic foot ulcer receiving optimization by plantar pressure measurements of their custom-made footwear and/or insoles	Process
E.3	Proportion of people with a (history of) diabetic foot ulcer treated within the context of a prevention management program for diabetic foot	Process

## Discussion

The aim of this study was to select evidence-based QIs for DFCs from a set of 42 candidate indicators identified from a systematic search in the literature (manuscript submitted for publication). A multidisciplinary stakeholder panel was asked to score the appropriateness of QIs based on their clinical judgement and guided by the collected supporting evidence, using the formal RAND/UCLA Appropriateness approach.

Of the 17 QIs rated as appropriate by the stakeholder panel in this study, 5 QIs addressed interventions that were not covered by the currently available QIs for diabetic foot care used in the different national initiatives on quality evaluation and monitoring.^16–18^ A first QI not considered so far was an indicator measuring the integration of a wound care specialty in the multidisciplinary team. This indicator resulted from the combination of two sets of candidate indicators that measured the treatment with non-biological dressings and the treatment with bioengineered skin substitutes. Interestingly, the stakeholders validated the use of such therapies, on the condition that it was delivered by a healthcare provider who would master their use, which constituted a shift from a process indicator to a structure indicator. This can be seen as a trade-off between the stakeholder acknowledgement of the potential of such emergent therapies to enhance wound healing versus the complexity of their use, and thus the requirement of adequate skills. A second QI not considered so far was an indicator that addresses the evaluation of the nutritional status of the patient. This indicator allowed to introduce the rising topic of the impact of malnutrition on DFU outcomes.^19^ Another QI not considered yet was an indicator which addresses the administration of low-density lipoprotein (LDL)-cholesterol-lowering medication, which indicates the effect of the lipid profile on the DFU patient^20^ and highlights the need for a more holistic view of the treatment. Two additional ‘new’ QIs addressed the implementation of care and prevention management protocols. This result underlines the fact that stakeholders believed that care structured by defined clinical management protocols indicates a good quality of care.

At the end of the selection process, an agreement could not be reached for seven indicators (A.5–B.4–B7a–B10b–B11a–B.11b–D.2). This concerned indicators for which the stakeholder panel seemed to have divergent opinions because of equipment accessibility, heterogeneity of interventions reported in the literature or issues to determine a specific population. However, since one of the objectives of the RAND/UCLA method is to bring out the points of discordance or indecision, these indicators were not rated again during phase 3, except the indicator addressing the application of knee-high offloading devices (D.2). Among the seven indicators, three had been rated as appropriate during phase 1. However, the opinions expressed or the modifications performed during the panel meeting in phase 2 influenced their final rating. For instance, the rating as uncertain of the indicator addressing the treatment with enzymatic debridement (B.11b) might be attributed to the introduction of a new indicator addressing the treatment with mechanical debridement (B.11c) in phase 2. Another notable case, for which a consensus could not be reached while it had been rated as appropriate during phase 1, was the indicator addressing the application of knee-high offloading devices (D.2). Together with the indicator addressing the application of non-removable knee-high offloading devices (D.1), this indicator was discussed again during phase 3 due to observed inconsistencies during analysis of phase 2. Finally, the indicator addressing the application of knee-high offloading devices (D.2) was rated as uncertain whereas the indicator addressing the application of non-removable knee-high offloading devices was rated as appropriate. In fact, these indicators were subjected to debate among the stakeholder panel, who highlighted the local realities (related to expertise or equipment availability) that make it difficult to apply such devices.

In our study, the selection of evidence-based QIs was conducted by using a formal and transparent methodology. Our approach, based on the RAND/UCLA Appropriateness method, relied on available scientific evidence, offered stakeholders a framework to discuss candidate QIs and included a quantitative method to measure their collective judgement. We recruited a panel representing the different disciplines active in diabetic foot care as well as a representative of the patient organization, which reflected the different expertise involved in the management of DFU. However, we could not recruit one Dutch-speaking and one French-speaking representative for each selected discipline. In addition, only Belgian stakeholders were included in the panel, which may limit the use of our results at an international level.

We complied with the main principle of the appropriateness method that consists of two separate, independent ratings in combination with a face-to-face stakeholder panel. An additional meeting had to be organized to resolve inconsistencies in the ratings observed in phase 2. Nevertheless, this did not impact the reliability and validity of our approach since recommendations to tackle such methodological issues have been provided by the developers of the method.^14^ In the healthcare domain, the RAND/UCLA Appropriateness method has been widely used within quality of care research to identify valid quality measures.^21–24^

The selection of evidence-based QIs was limited by the fact that supporting high-quality evidence was not available for some QIs. However, this is what stakeholder panels/consensus methods are dedicated to. When the highest level of evidence is not available, they aim to identify the processes of care that are most likely to be valid measures of quality.

Predictably, most of the QIs rated as appropriate addressed interventions which are commonly endorsed by the international guidelines of diabetic foot care.^25–28^ This was logical since guidelines are also based on evidence and our panel of healthcare providers know the guidelines and put them into practice. Nevertheless, our use of an open-minded literature review to identify QIs rather than guidelines offers additional input. The use of literature, instead of guidelines, not only brings new topics for QIs but also allows reflection on the feasibility of an indicator, regardless if the intervention has been recommended or not.

The stakeholder panel did not feel obliged to accept the measure of an intervention because that intervention was endorsed by guidelines. They could put their judgement in perspective of their daily practice and the provided objective evidence.

In conclusion, we report the selection of a set of 17 evidence-based QIs for diabetic foot care by a multidisciplinary group of stakeholders from DFU care. We used a reliable methodology to fill the gaps identified in the development of existing QIs. Several indicators were introduced that were not previously described. The identified evidence-based QIs offer an open-minded view of the measures that can be used in DFCs to monitor and evaluate quality of care. In this study, we did not intend to question well-accepted QIs but rather to reinforce them and offer new evidence-based structure and process indicators. Further work is needed to evaluate the complementarity of these QIs with the existing QIs and their implementation in clinical practice.

## Supplementary Material

ckad235_Supplementary_Data

## Data Availability

The data that support the findings of this study are available from the corresponding author, FML, upon reasonable request. Key points There is a need for valid quality indicators to assess quality of care delivered to DFU patients. A multidisciplinary panel of stakeholders from Belgian DFU care identified relevant and feasible evidence-based quality indicators, using a modified Delphi consensus. The identified evidence-based QIs provide an assessment tool to evaluate and monitor quality of care delivered to DFU patients. Strategies should be undertaken to facilitate the implementation of these QIs in diabetic foot clinics.
