# Supplementary material for: A multidisciplinary Delphi consensus to define evidence-based quality indicators for diabetic foot ulcer care
Source: Eur J Public Health. 2024 Jan 26;34(2):253–9. doi: 10.1093/eurpub/ckad235 (PMC10990505; doi:10.1093/eurpub/ckad235)
Supplement: ckad235_Supplementary_Data [file ckad235_supplementary_data.pdf]

Supplementary data

Table S1. List of 42 candidate QIs provided to experts during the consensus process

No.	Indicator	Indicator type
	Domain: Organization of care	
A.1	Proportion of people with a diabetic foot ulcer receiving multidisciplinary foot care	Structure
A.2	Proportion of people with a diabetic foot ulcer receiving multidisciplinary foot care with an integrated podiatric specialty	Structure
A.3	Proportion of people with a diabetic foot ulcer treated within the context of a care management program for diabetic foot	Structure
A.4	Proportion of diabetic foot clinics that participate to a pay-for-performance program	Structure
A.5	Proportion of people with a diabetic foot ulcer receiving nurse-led care	Structure
	Domain: Wound healing interventions	
B.1a	Proportion of people with a non-healing diabetic foot ulcer treated with non-biological dressings (umbrella indicator[a])	Process
B.1b	Proportion of people with a non-healing diabetic foot ulcer treated with non-biological dressings impregnated with antimicrobial agents[b]	Process
B.1c	Proportion of people with a non-healing diabetic foot ulcer treated with non-biological dressings not impregnated with antimicrobial agents[b]	Process
B.2a	Proportion of people with a non-healing diabetic foot ulcer treated with a bioengineered skin substitutes (umbrella indicator[a])	Process
B.2b	Proportion of people with a non-healing diabetic foot ulcer treated with acellular dermal matrix	Process
B.2c	Proportion of people with a non-healing diabetic foot ulcer treated with allogeneic skin substitute	Process
B.2d	Proportion of people with a non-healing diabetic foot ulcer treated with autologous skin substitute	Process
B.3	Proportion of people with a non-healing diabetic foot ulcer treated with isolated cellular therapy	Process
B.4a	Proportion of people with a diabetic foot ulcer treated with systemic hyperbaric oxygen therapy	Process
B.4b	Proportion of people with a diabetic foot ulcer and adequate perfusion treated with systemic hyperbaric oxygen therapy	Process
B.4c	Proportion of people with a diabetic foot ulcer and inadequate perfusion treated with systemic hyperbaric oxygen therapy	Process
B.5	Proportion of people with a non-healing diabetic foot ulcer treated with isolated growth factor	Process
B.6	Proportion of people with a non-healing diabetic foot ulcer treated with negative pressure wound therapy	Process
B.7a	Proportion of people with a non-healing diabetic foot ulcer treated with laser/phototherapy	Process
B.7b	Proportion of people with a non-healing diabetic foot ulcer treated with extracorporeal shockwave therapy	Process
B.7c	Proportion of people with a non-healing diabetic foot ulcer treated with ultrasound therapy	Process
B.7d	Proportion of people with a non-healing diabetic foot ulcer treated with physical therapy other than laser, shockwave or ultrasound	Process
B.8a	Proportion of people with a non-healing diabetic foot ulcer treated with topical oxygen therapy	Process
B.8b	Proportion of people with a non-healing diabetic foot ulcer treated with ozone therapy or combined oxygen-ozone therapy	Process
B.9a	Proportion of people with a non-healing diabetic foot ulcer treated with a single nutrient supplementation	Process
B.9b	Proportion of people with a non-healing diabetic foot ulcer treated with a multi-nutrient supplementation	Process
B.10a	Proportion of people with a non-healing diabetic foot ulcer treated with pharmacological agents having an action on vessels	Process
B.10b	Proportion of people with a non-healing diabetic foot ulcer treated with pharmacological agents having an action on immunity	Process

B.11a	Proportion of people with a non-healing diabetic foot ulcer treated with biological debridement	Process
B.11b	Proportion of people with a non-healing diabetic foot ulcer treated with enzymatic debridement	Process
B.12a	Proportion of people with a non-healing diabetic foot ulcer treated with amputation	Process
B.12b	Proportion of people with a non-healing diabetic foot ulcer treated with bony surgical offloading	Process
B.12c	Proportion of people with a non-healing diabetic foot ulcer treated with soft tissue surgical offloading	Process
Domain: Peripheral artery disease (PAD)		
C.1a	Proportion of people with a diabetic foot ulcer and inadequate perfusion treated with endovascular surgery	Process
C.1b	Proportion of people with a diabetic foot ulcer and inadequate perfusion treated with open vascular surgery	Process
C.1c	Proportion of people with a diabetic foot ulcer and inadequate perfusion undergoing revascularization based on the angiosome concept	Process
Domain: Offloading		
D.1	Proportion of people with a non-infected, non-ischemic plantar neuropathic diabetic foot ulcer treated with a non-removable knee-high offloading device	Process
D.2	Proportion of people with a non-infected, non-ischemic plantar neuropathic diabetic foot ulcer treated with a knee-high offloading device	Process
Domain: Secondary prevention		
E.1	Proportion of people with a (history of) diabetic foot ulcer receiving patient education	Process
E.2a	Proportion of people with a history of peripheral neuropathy (PNP) receiving therapeutic footwear and/or custom-made insoles, or custom-made shoes	Process
E.2b	Proportion of people with a history of diabetic foot ulcer receiving optimization by plantar pressure measurements of their custom-made footwear and/or insoles	Process
E.3	Proportion of people with a (history of) diabetic foot ulcer treated within the context of a prevention management program for diabetic foot	Process

[a] umbrella indicator = unifying indicator under which the specific and related interventions was grouped and which allows to assess the delivery of such therapy regardless the type.

[b] honey derivatives, silver or antibiotics

Table S2. Final set of QIs classified as inappropriate

No.	Indicator	Indicator type
Domain: Organization of care		
A.4	Proportion of diabetic foot clinics that participate to a pay-for-performance program	Structure
Domain: Wound healing interventions		
B.3	Proportion of people with a non-healing diabetic foot ulcer treated with isolated cellular therapy	Process
B.5	Proportion of people with a non-healing diabetic foot ulcer treated with isolated growth factor	Process
B.7b	Proportion of people with a non-healing diabetic foot ulcer treated with extracorporeal shockwave therapy	Process
B.7c	Proportion of people with a non-healing diabetic foot ulcer treated with ultrasound therapy	Process
B.7d	Proportion of people with a non-healing diabetic foot ulcer treated with physical therapy other than laser, shockwave or ultrasound	Process
B.8a	Proportion of people with a non-healing diabetic foot ulcer treated with topical oxygen therapy	Process
B.8b	Proportion of people with a non-healing diabetic foot ulcer treated with ozone therapy or combined oxygen-ozone therapy	Process

Table S3. Final set of QIs for which a collective opinion could not be reached

No.	Indicator	Indicator type
Domain: Organization of care		
A.5	Proportion of people with a diabetic foot ulcer receiving nurse-led care	Structure
Domain: Wound healing interventions		
B.4	Proportion of people with a diabetic foot ulcer treated with systemic hyperbaric oxygen therapy	Process
B.7a	Proportion of people with a non-healing diabetic foot ulcer treated with laser/phototherapy	Process
B.10b	Proportion of people with a non-healing diabetic foot ulcer treated with pharmacological agents having an action on immunity	Process
B.11a	Proportion of people with a non-healing diabetic foot ulcer treated with biological debridement	Process
B.11b	Proportion of people with a non-healing diabetic foot ulcer treated with enzymatic debridement	Process
Domain: Offloading		
D.2	Proportion of people with a non-infected, non-ischemic plantar neuropathic diabetic foot ulcer treated with a knee-high offloading device	Process
